# Application of Strain Engineering in Solar Cells

**DOI:** 10.3390/molecules29143260

**Published:** 2024-07-10

**Authors:** Houzhi Fei, Caiyi Shang, Dandan Sang, Changxing Li, Shunhao Ge, Liangrui Zou, Qinglin Wang

**Affiliations:** School of Physics Science and Information Technology, Liaocheng University, Liaocheng 252000, China; lcufeihouzhi@163.com (H.F.); 2021403177@stu.lcu.edu.cn (C.S.); 17853058528@163.com (C.L.); geshunhao223@163.com (S.G.); zouliangruilcu@163.com (L.Z.)

**Keywords:** solar cell, strain engineering, efficiency, stability, strain regulation, power conversion efficiency

## Abstract

Solar cells represent a promising innovation in energy storage, offering not only exceptional cleanliness and low cost but also a high degree of flexibility, rendering them widely applicable. In recent years, scientists have dedicated substantial efforts to enhancing the performance of solar cells, aiming to drive sustainable development and promote clean energy applications. One approach that has garnered significant attention is strain engineering, which involves the adjustment of material microstructure and organization through mechanical tensile or compressive strain, ultimately serving to enhance the mechanical properties and performance stability of materials. This paper aims to provide a comprehensive review of the latest advancements in the application of strain engineering in solar cells, focused on the current hot research area—perovskite solar cells. Specifically, it delves into the origins and characterization of strain in solar cells, the impact of strain on solar cell performance, and the methods for regulating stable strain. Furthermore, it outlines strategies for enhancing the power conversion efficiency (PCE) and stability of solar cells through strain engineering. Finally, the paper conducts an analysis of the challenges encountered in the development process and presents a forward-looking perspective on further enhancing the performance of solar cells through strain engineering.

## 1. Introduction

Given the dwindling reserves of fossil fuels and the environmental degradation resulting from their consumption, traditional energy sources are increasingly unable to support the demands of future sustainable growth [1]. Photovoltaic technology, particularly solar cells, presents a renewable energy alternative capable of directly transforming photon energy into electrical power in an eco-friendly manner. The quest for photovoltaic devices that are both highly efficient and cost-effective for mass production persists, despite the lack of complete success in this endeavor. As solar cell technology gains global traction, there is a pressing need to refine the associated materials and devices to slash production costs and elevate performance [2]. At the laboratory level, solar cells have achieved a maximum PCE surpassing 26%, with further enhancement anticipated [3,4,5,6,7,8]. Solar cells are mainly divided into three categories: silicon-based solar cells, thin film solar cells, and new materials solar cells. At present, the efficiency of silicon-based solar cells has reached 26.7% [9], with a very high market share, but the manufacturing cost is high, and the efficiency improvement space is limited. Thin film solar cells can effectively overcome the cost, weight, and scalability problems in silicon-based solar cells [10], with low manufacturing costs, light weight, and other advantages, but relatively low efficiency. New material-based solar cells have recently attracted great attention, such as the perovskite solar cells, and although they show high efficiency under laboratory conditions, they still face challenges in terms of stability and large-scale production [11,12,13,14]. Strain engineering has emerged as a versatile strategy for material regulation. It offers potential improvements in band structure and photovoltaic properties [15,16,17,18], mitigates degradation mechanisms [19,20], and alleviates residual strain [21,22,23,24,25,26], thereby charting a novel course for property enhancement [27].

Strain, which refers to the deformation experienced by the lattice constants of ideal crystal structures due to intrinsic distortions (like point defects or orientation disparities) or external stresses (such as those induced by light, heat, or pressure), plays a crucial role in determining the photovoltaic characteristics and inherent stability of polycrystalline materials [28]. Strain sources can be categorized into externally induced strain and local lattice strain, with origins ranging from thermal expansion coefficient mismatch or lattice constant mismatch [29,30,31,32], ion mismatch [33], lattice distortions and fractures [34], radiation damage [35], etc. Strain engineering can control the material phase transition, which greatly overcomes the degradation of the whole equipment performance due to local defects. The mitigation of adverse conditions caused by lattice mismatches, crystal distortions, and thermal expansion mismatches has greatly increased the stability of perovskite solar cells and opened the way for the realization of large area perovskite solar cells (PSCs) [36]. In addition, thanks to the effective regulation of strain on material properties, the ability to replace traditional high-cost materials with cheaper materials has become a reality, greatly reducing the manufacturing costs. Research by Wu et al. [37] found that bias voltage can promote ion migration and accumulate interfacial stress, resulting in lattice distortion or atomic bond breakage, resulting in interfacial defects. This can be regulated by constructing a stress buffer layer (such as a fullerene derivative) at the interface, which provides a solution for suppressing lattice strain and improving stability. Li et al. [38] endowed PSCs with stretchability through out-of-plane deformation design, which significantly reduced internal stress and greatly improved the tensile, torsional, and bending properties of PSCs. Therefore, it is evident that the stabilization and optimization of internal and external stress/strain is a powerful means to solve the common problems of inefficiency and instability. 

**Figure 1:**
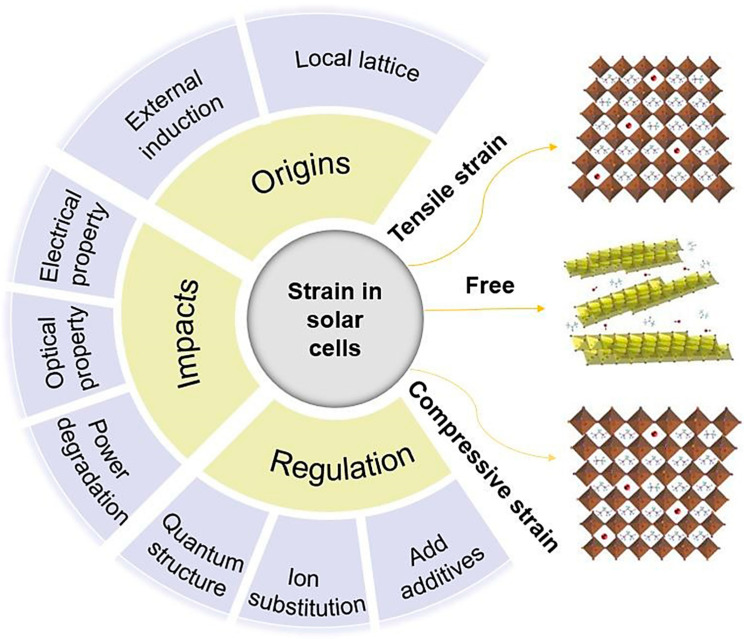
In this paper, the origin, impact, and regulation strategy of the strains in solar cells were discussed.

In recent years, the field of solar cells has made rapid development; however, regarding “how to use strain engineering to improve the efficiency and stability of solar cells”, there is still a lack of systematic summary, especially in the field of perovskite solar cells. This paper discusses the origin of strain and highlights recent breakthroughs in the effects of strain engineering on solar cells, including changes in electronic band structure, optical and electrical properties, power degradation, trap state density, ion migration, and photoinduced stability. Strategies to enhance the performance of solar cells through strain engineering (Figure 1), such as the use of III–V compounds to create quantum dots or quantum wells, are highlighted. The challenges and future prospects of strain engineering in the field of solar cell application are summarized in order to promote the key role of strain engineering in the design and optimization of photovoltaic devices.

## 2. Origin of Strain in Solar Cells

Strain instability of materials and devices is a key problem in the development of solar cells. This strain usually results from a variety of factors, including the thermal expansion properties of the material, microstructure defects, and layer mismatches in the manufacturing process and so on. In order to create more stable solar cells, the sources of these strains must first be deeply understood and quantified [13].

### 2.1. External Conditions Induce Strain

Externally induced strain can be caused by factors such as the type and content of ions in the composition, biaxial strain, thermal expansion mismatch, and light and bias induction. Among these, thermal expansion mismatch is an important factor. The various materials used in solar cells, such as metals, semiconductors, and substrates, have different coefficients of expansion. When a solar cell goes through a thermal cycle, due to the different coefficient of thermal expansion of materials such as metals, semiconductors, and substrates, they expand at different rates when the temperature increases and contract at different rates when the temperature decreases. This difference in expansion and contraction causes stresses and strains between materials. The continuous accumulation of strain may lead to the deformation, fracture, or performance degradation of the material. Heterovalent metal (such as Bi^3+^) ions are incorporated into perovskites in an attempt to induce electron doping and increase the carrier density in the semiconductor. Unfortunately, this inevitably creates strain, causing defect states and increasing non-radiative recombination. Therefore, the reasonable regulation of strain has a crucial impact on the photovoltaic properties of solar cells [39]. When studying the structural dynamics and photoelectrical properties of FAPbI_3_ under tensile strain, it was found that the tensile strain resulted in longer Pb-I bonds and less inclined PbI_6_ octahedra. The expansion of the lattice makes the bandgap increase and the valence band maximum decrease, which can affect the generation and collection of charge carriers in the interface by changing the interface. In addition, the tensile strain reduces the electron–hole recombination rate in FAPbI_3_. This study shows that the photovoltaic properties of the solar cell can be effectively regulated by strain [40]. The non-uniform strain of the perovskite layer coated on the substrate can lead to the formation of defects at the grain boundaries. Grain boundary passivation was successfully achieved by adding a certain amount of trioctylphosphine (TOP) to the MACL-containing FAPbI_3_ pryecursor solution, which reduced external strain and significantly improved the performance of the PSCs [41].

**Figure 2:**
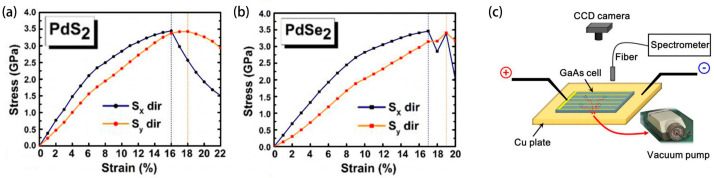
Illustrates the relationship between stress (GPa) and strain (%) under tensile conditions for both penta-PdS_2_ (**a**) and penta-PdSe_2_ (**b**) (reprinted with permission from Ref. [39], copyright 2022, Springer Nature). (**c**) Schematic diagram of the EL measuring device (reprinted with permission from Ref. [43], copyright 2018, Elsevier).

Raval et al. [42] found that the conversion efficiency of monolayer penta-PdQ_2_ films, where Q represents either sulfur (S) or selenium (Se), could be modulated by applied strain. Their investigations revealed that a monolayer of penta-PdS_2_ is capable of withstanding mechanical stresses of up to 16% and 18% along the X and Y axes, respectively, as depicted in Figure 2a. Similarly, the penta-PdSe_2_ monolayer exhibited resilience to strains of 17% and 19% along the same axes (Figure 2b). Notably, under compressive strain conditions, certain levels of strain led to an increase in efficiency beyond the baseline value, achieving 33.93% for penta-PdS_2_ with −5% compressive strain and −33.94% for penta-PdSe_2_ with a −2% compressive strain. Contrastingly, when subjected to tensile strain, both penta-PdS_2_ and penta-PdSe_2_ experienced a decrease in efficiency to 25.72% and 33.63%, respectively, with +1% tensile strain, falling below the initial efficiency. These findings indicate that the Shockley–Queisser (SQ) efficiency can be effectively tuned through the application of biaxial strain to penta-PdQ_2_ monolayers. Such tunability is critical for optimizing the performance of solar cells. Moreover, the fabrication of solar cells based on penta-PdQ_2_ offers a promising avenue for enhancing photovoltaic efficiency when compared to conventional silicon-based counterparts. The exceptional thinness of PdQ_2_ monolayers positions them as promising candidates for the next generation of solar cell technologies and advanced nanodevices.

Electroluminescence (EL) imaging technology can detect the externally induced strain of gallium arsenide thin film solar cells. Figure 2c illustrates the schematic diagram of the EL measurement setup. The investigation revealed that the strained region not only amplified the local luminescence intensity but also caused a redshift in the luminescence peak. Specifically, under tensile strain, the bandgap decreases, with a reduction significantly greater than the increase observed under compression strain, and the optical hole guide moves towards the band direction (opposite behavior occurs under compression strain). The model takes into consideration the external biaxial strain and establishes a relationship between the energy displacement in the energy spectrum and the strain. Finally, an approximation was made to determine the spatially resolved tensile strain of the EL images by correlating the emission intensity of EL with the spectral displacement and external strain. This study offers a straightforward approach for diagnosing externally induced strain in gallium arsenide thin film solar cells using EL measurements [43].

Oksenberg and colleagues [44] implemented an investigation into the substantial size-dependent photoluminescence noted in stable monocrystalline cesium lead bromide (CsPbBr_3_) nanowires by constructing an ordered assemblage. Through the application of nanowire ensembles of variant lengths, as well as a solitary conical nanowire exhibiting a notable axial gradient during growth, they deduced the presence of pronounced emission reliance upon spatial displacement. Photoluminescence excitation (PLE) experimentation revealed that variations within the nanowires’ bandgap primarily catalyze this effect, disproving reabsorption as the mechanism behind the observed bandgap alteration. Analysis utilizing scanning transmission electron microscopy (STEM), scanning electron diffraction (SED), and scanning X-ray diffraction indicated that strain generated by heteroepitaxial processes, alongside lattice relaxation, fostered extensive lattice rotations within the nanowire cross-sections. Consequently, these distortions govern the bandgap evolution and facilitate an energetic ascension of the emission spectrum with diminishing nanowire height. Identifying the strains evolved from lattice reactions to heteroepitaxy broadens our grasp of metal halide perovskites (MHPs), aiding in their refinement for enhanced device potential.

In the realm of photovoltaics, organic–inorganic metal halide perovskites have garnered considerable scrutiny due to their swiftly ascending power conversion efficiencies. Research by Kim et al. [45] scrutinized the influences of illumination and electrical bias on perovskite structural integrity, uncovering periodicity in fringed ferroelastic domains within the grains, which are highly amenable to such external forces. The application of the Williamson–Hall (W-H) analysis on X-ray diffraction data traced the source of these domain shifts to the applied stimuli. The team proposed that these domains could establish pathways conducive to hole transport under positive bias, potentially elucidating hysteresis observed in the current–voltage characteristics of halide perovskite solar cells (PSCs). These insights could underpin further exploration into nanoscale structural impacts on solar cell efficacy and the fine-tuning of perovskite photovoltaic modules. In the process of high temperature annealing, defects and strains of perovskite films are inevitably produced. The in situ self-polymerization (ISP) strategy is a good solution. By adding a self-polymerizable N-methylacrylamide (NMA) monomer to the perovskite film, the thermal expansion of perovskite during the thermal crystallization process was successfully limited, effectively releasing 34% of the residual tensile strain in the perovskite film. The optimized device achieved a champion PCE of 22.9%, while the unoptimized device achieved a PCE of only 20.9%. In environmental stability tests, devices without NMA retained only about 69% of their initial PCE after 1500 h of storage, and devices with NMA retained about 91% of their initial PCE value [46]. The introduction of multi-active-site additive pemirolast potassium (PP) into perovskite films can effectively improve carrier dynamics and release residual stress. This can be attributed to the interaction between PP and perovskite molecules to improve the crystallization and quality of perovskite films. The residual stress on the surface of the modified film is effectively released by 30%. The average carrier lifetime increased significantly from 392.39 ns to 638.65 ns, and the PCE increased by about 10%. In thermal stability tests, the modified device retained 90% of the initial PCE, while the control device retained only 67%. It can be seen that the adsorption of multiple active sites is more favorable than that of single active site [18]. 

Elsewhere in solar technology advancements, Chen et al. [47] investigated the strain engineering potential within alpha-formamidine lead iodide (alpha-FAPbI_3_). Through precision in substrate lattice calibration, epitaxial growth was achieved, imposing an upward of 2.4% compressive strain on the α-FAPbI_3_ film. The investigation found strain-induced shifts in the crystal structure, which manifested as a narrowed bandgap and heightening of hole mobility. Moreover, strain epitaxy demonstrated notable phase stability for α-FAPbI_3_, attributable to the combined influence of epitaxial stabilization and strain compensation.

### 2.2. Local Lattice Strain

Local lattice strain refers to the deformation that arises from slight inconsistencies in the lattice structure between a specific area and its surrounding regions within a crystalline material. Li et al. [48] analyzed the heterogeneous structure of perovskite films through nano-focused wide-angle X-ray scattering, revealing differences in the characteristic peaks. These discrepancies pointed to tensile strain in the upper portion of the film, indicating a higher rate of vertical grain growth. This observation suggests that the direction of rapid vertical growth dictates the final preferred orientation of the perovskite grains. Furthermore, the researchers discovered that water-induced degradation typically occurs at the perovskite–air interface and along lateral grain boundaries. The study also highlighted that the tensile strain on the upper surface was a significant factor contributing to wet degradation. These findings offer a deeper understanding of the local crystal structure of perovskite thin films and provide valuable insights for advancing the development of PSCs.

In general, a variety of strategies can be adopted to control and optimize the various sources of strain during the design and manufacturing of solar cells. The effect of nanoscale structural modification on the performance of solar cells has produced unexpected effects, which can be further explored to promote the optimization of the performance of photovoltaic devices. The attainment of phase stability is realizable by the concerted action of epitaxial growth coupled with compressive strain. Furthermore, the degradation of PSCs is primarily attributable to the tensile strain present on the uppermost layer. The local crystal structure of perovskite thin films should be further studied to promote the development of perovskite nanomaterials. The performance of solar cells can be affected by both external condition-induced strain and local lattice strain; thus, it is essential to control the strain when preparing solar cells. In the following text, the effect of strain on solar cells and the use of strain engineering strategies to control performance will be discussed in detail.

## 3. Characterization of Strain in Solar Cells

**Figure 3:**
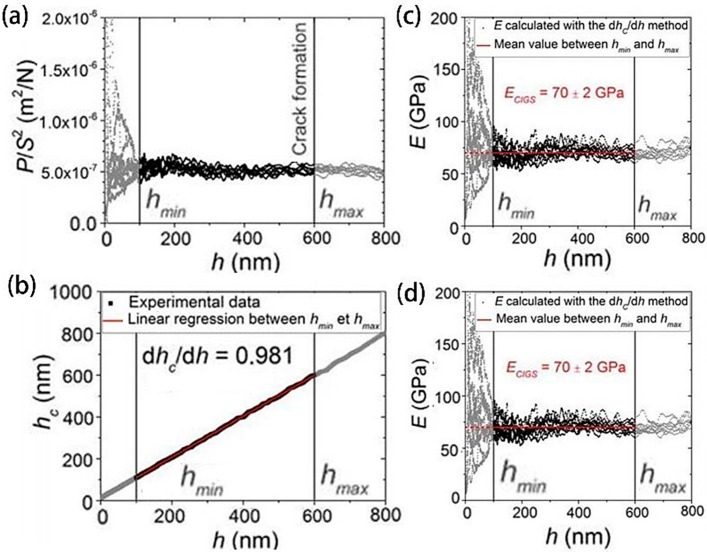
(**a**) The square load of the contact stiffness ratio. (**b**) Depiction of the contact depth. (**c**) The apparent Young’s modulus. (**d**) The change in the apparent hardness corresponding to the depth of penetration. (Reprinted with permission from Ref. [50]. Copyright 2022, American Association for the Advancement of Science).

Employing pulsed light excitation as a powerful observational tool, the multifaceted structural and electronic interactions within layered 2D halide perovskites can be scrutinized. In their study, Fu et al. [49] deployed transient reflection spectroscopy (TRS) to unravel the intrinsic dynamics of strain propagation in two-dimensional perovskite single crystals. They posited that the genesis, dissemination, and detection of strain in a typical 2D perovskite framework is largely unimpeded by the nature of the substrate or the presence of multiple phases. By harnessing Brillouin scattering, the research team was able to discern strain pulses resulting from the interaction between thermoelastic stress and disparate lattice states. A collaborative approach utilizing a dual-temperature model with strain wave propagation analysis revealed that both thermoelastic and disparate lattice factors are pivotal in strain evolution. They believe that due to the weak van der Waals bonds between organic layers, the out-of-plane lattice stiffness is reduced, resulting in slower strain propagation. The insight paves the way towards a nuanced comprehension of fundamental strain characteristics present in layered perovskites, opening new avenues for fine-tuning their operational properties for innovative applications. In a related vein, Gerthoffer et al. [50] conducted measurements and comparisons of Young’s modulus (E) and hardness (H) properties of CIGS deposited on Mo coated SLG and Mo coated UTG using the nanoindentation method. Their findings indicated E = 70 ± 2 GPa and H = 3.4 ± 0.1 GPa for the CIGS on Mo/UTG (as depicted in Figure 3a–d), and E = 68 ± 2 GPa, H = 3.0 ± 0.1 GPa for the CIGS on Mo/SLG. The significance of this methodology extends to evaluating the mechanical integrity of other pliable thin-film photovoltaic systems, such as amorphous silicon, cadmium telluride, and perovskite. Further numerical simulations addressing the bending-induced strains in CIGS solar cells indicated a correlation between the strain manifestation and the neutral axis’ placement within the curved structure. On the convex side, tensile strain accumulates, whereas the concave side is subjected to compressive strain. The studies also deduced that substrates characterized by a lesser thickness and low Young’s modulus are effective in alleviating the film’s strain for a specified curvature radius.

## 4. Effect of Strain on Solar Cells

The effects of strain on the structure and performance of solar cells are then discussed. Here, we mainly discuss the following aspects: electronic band structure, optical and electrical properties, power degradation, trap state density, ion migration, and thermal equilibrium.

### 4.1. Electron Band Structure

Strain energy directly influences the energy distribution of solar cell materials. Compressive strain may lead the top of the valence band to shift to a higher energy level, while tensile strain may cause the bottom of the conduction band to move downward. This changes the bandgap width of the material, significantly affecting the absorption spectrum, as well as carrier generation and recombination dynamics. Notably, strain engineering is identified as a useful strategy for modulating electronic properties by adjusting the band structure of a crystal [51]. 

Antimony trisulfide (Sb_2_S_3_), a plentiful natural resource, shows potential for harnessing solar energy and heat flow. Cui et al. [52] studied the response of the layered structure of Sb_2_S_3_ to external stresses and revealed that at 20 GPa, (Sb_4_S_6_)_n_ completely collapsed. Their detailed structural analysis identified two crucial transformations occurring at approximately 4 GPa and 11 GPa. The direct bandgap pattern emerges at around 4 GPa, while a fully developed three-dimensional Sb_2_S_3_ crystal structure forms above 11 GPa. The engineering of the bandgap in Sb_2_S_3_ is characterized by a biphasic transitional process. Initially, the transition results from the band state redistribution near the maximum conduction band. Subsequently, the second phase transition is dictated by the isomorphic phase transition, i.e., the layer collapse. This mechanism masks the evolution of the bandgap energy in compressed Sb_2_S_3_. Although compression alone cannot yield strong light luminescence, future studies could explore a combination of doping and pressurization to optimize the band structure. This gradient bandgap proves advantageous in device engineering. Subsequent investigations revealed that the gradient of the bandgap, induced by strain, escalates in conjunction with an increase in defect density. However, it was observed that the bandgap grading is counterbalanced by the amplified presence of defects, leading to a performance parity between strained and unstrained PSCs when subjected to standard one sun illumination conditions. However, this compensation does not occur in low light conditions, and the increased defect density accelerates the device degradation process. Thus, the negative effects of increased defect density should be carefully considered in practical applications [53].

**Figure 4:**
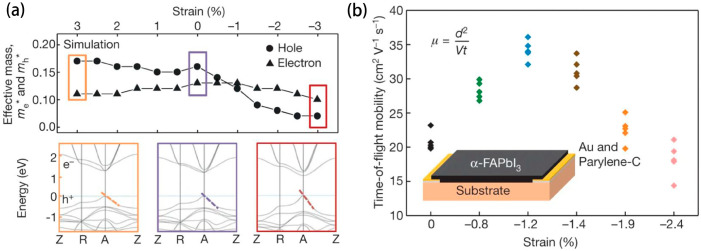
(**a**) Effective mass of the carrier under different strains (above). Electron band structure at three strain levels (3%, 0% and −3%) (bottom). (**b**) The relationship between carrier mobility and strain magnitude (color symbols only represent strains of different sizes). (Reprinted with permission from Ref. [48]. Copyright 2020, Springer Nature).

The effect of strain on bandgap and carrier mobility cannot be ignored. For the bandgap, the compression strain will reduce the atomic spacing and enhance the interaction between atoms, resulting in the band width and bandgap reduction. This effect is more pronounced in some semiconductor materials, such as silicon and germanium. On the contrary, tensile strain will increase the atomic spacing, weaken the interatomic interaction, and lead to the narrowing of the band and the widening of the bandgap. This effect may be more significant in some wide-gap semiconductor materials [36]. Figure 4a illustrates this in detail. In addition, strain can affect carrier mobility by changing the material’s lattice structure and electron scattering mechanism. Compressive strain typically increases lattice defects and scattering centers in the material, resulting in enhanced scattering of carriers and thus reduced carrier mobility. The tensile strain may reduce the lattice defects and scattering centers, weaken the scattering of carriers, and thus increase the carrier mobility [17]. Figure 4b shows the variation of carrier mobility with the magnitude of strain [48].

### 4.2. Optical and Electrical Properties

The strain not only changes the band structure but also affects the dielectric function and the effective mass of the carrier. The section examines the effect of strain on the photoelectrical characteristics of solar cells. It involves variables such as the open circuit voltage (*V*_OC_), fill factor (FF), short circuit current (*I*_SC_), spectral responsiveness, and overall energy transformation efficiency. The study shows that modifications in these attributes markedly influence the solar cell’s design refinement process [54]. 

**Figure 5:**
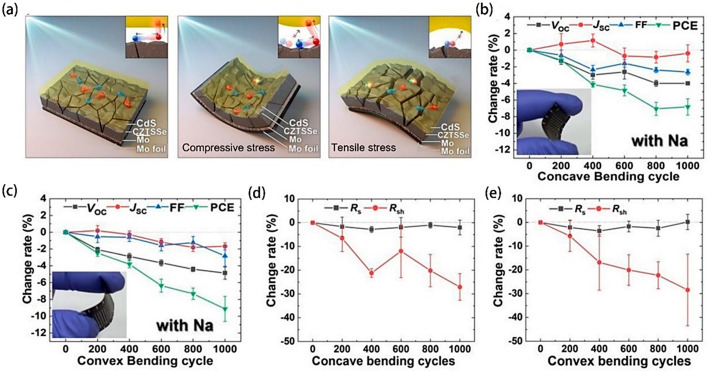
(**a**) Illustrates grain boundary maps of polycrystalline CZTSSe under varying mechanical stress—namely flat, concavely bent, and convexly bent configurations. Electrons are depicted as red spheres while holes are blue. With a bending radius set at 50 mm and a maximum flexure event count of 1000, (**b**,**c**) delineates the variation rate in photovoltaic device parameters during concave and convex bending, respectively, while (**d**,**e**) quantify the change rate in series and parallel resistances under identical bending conditions, corresponding to concave and convex deformation. (Reprinted with permission from Ref. [56]. Copyright 2022, Springer Nature).

An innovative component for semiconductor-sensitized solar cells (SSSCs) was recently discovered, utilizing a TiO_2_ backbone with CdSe nanosheets (NPLs) layered on top and a ZnS coating. The composite TiO_2_/CdSe NPLs/ZnS electrode is sought after for its superior charge transport and light capture efficiencies, as evidenced by the redshift in both absorption and photoluminescence spectra, together with reduced luminescence lifetimes. These enhancements are attributed to the type II band alignment between the CdSe NPLs and ZnS, fostering photodipole generation. Studies of CdSe/ZnS core–shell quantum dots show that lattice strain is caused by lattice mismatch (12%) between CdSe and ZnS. This creates a possible dislocation defect at the interface, which ultimately results in a decrease in quantum yield. The revolutionary NPL-based sensitized solar cell manifests a peak *V*_OC_ of 664 mV and an optimal *I*_SC_ reaching 11.14 mA/cm^2^, cumulatively contributing to a conversion efficiency of 1.94%. Nevertheless, further augmentations remain feasible—for instance, refining the TiO_2_ layering technique or enhancing NPL deposition could heighten NPL content, bolster absorption efficacy, and suppress recombinative actions inside the cell. [55]. Park et al. [56] scrutinized Cu_2_ZnSn(S,Se)_4_ (CZTSSe) optoelectronic constructs under the influence of mechanical pressures and conceived highly efficient, pliable CZTSSe devices by modulating sodium incorporation. Employing band structure diagram for CZTSSe in various configurations (Figure 5a)—plane, concave, and convex bent shapes—they demonstrated that the deformation of the electronic structure due to stress impacts band alignment at interfaces and, thus, charges carrier dynamics. A deeper foray into electrical characteristics unveiled that mechanical strain results in a diminished electron barrier at grain junctures, leading to weaker carrier segregation and heightened recombination (Figure 5b,c). Measurements using surface photovoltage (SPV) techniques revealed increased local *V*_OC_ losses under strain, which are particularly more pronounced in convex curves, hinting at enhanced carrier recombination. Consequently, the deterioration in carrier movement was notably more significant in convex compared to concave bends. The degradation in Na-doped CZTSSe was less pronounced, with parallel resistance decay after a thousand bending cycles being substantially greater than that of the series resistance (Figure 5d,e).

**Figure 6:**
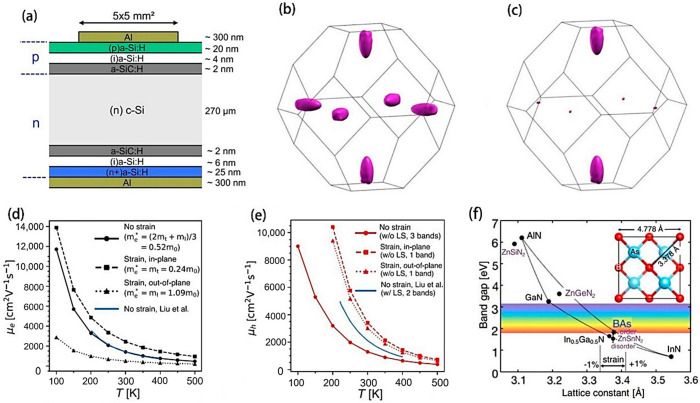
(**a**) Architecture of a silicon heterojunction solar cell (reprinted with permission from Ref. [57], copyright 2020, Elsevier). The isosurfaces of the lowest conduction band of unstrained BAs (**b**) and BAs under 1% tensile strain (**c**) are situated 200 meV above the CBM within the first Brillouin zone (BZ). The temperature-dependence of carrier mobility in BAs for both electrons (**d**) and holes (**e**) subjected to tensile strain, respectively [61]. (**f**) The relationship between bandgap and in-plane lattice constants of BAs, wurtzite group III nitrides and orthogonal ZN-IV nitrides (reprinted with permission from Ref. [59], copyright 2020, Springer Nature).

Guin et al. [57] documented the effects of diverse uniaxial tensile stress magnitudes on the dark current–voltage relationships inherent to silicon heterojunction (SHJ) solar cells (Figure 6a). They detected a reversible influence on these characteristics and proposed a dual-exponential model to characterize the impact of strain on the diffusion saturation current. It was observed that the diffusion saturation current exhibited a decrement upon attaining certain strain thresholds, particularly a notable reduction of approximately 3% at a longitudinal strain of 6.7 × 10^−4^. Through comparison with the theoretical model, it was found that the theoretical prediction agrees well with the experimental results, but there are differences when considering the effect of strain on valence band density. The research conclusively showed the reversibility of uniform strain impacts on the dark I-V characteristics of solar cells. Nevertheless, it should be noted that while their investigation was restricted to SHJ cells with relatively thicker silicon substrates, the implications of strain might be more pronounced in thinner crystalline silicon solar cells and this opens an avenue for further exploration. Smith et al. [58] first discovered the behavior of (CdSe)ZnS, (CdSe)CdS, and other quantum heterostructures in response to strain. The standard type-I quantum dot behavior is replaced by type-II quantum-dot behavior, which leads to the spatial separation of electrons and holes, an extended excited state lifetime, and a large spectral shift. This research will be important for multiexciton generation and efficient solar energy conversion.

In investigating the effect of biaxial strain on the heterostructure of boron arsenide, the authors found that the tensile strain causes a triple degenerate lift in the conduction band (Figure 6b,c), and that at 1% strain, the out-of-plane minimum is about 200 meV lower than the two in-plane minimums. Such disparity in energy introduced by 1% biaxial tensile strain profoundly enhances the carrier mobility within the crystal plane, portending advancements in switch operation rapidity and transistor power efficacy. The investigation into the thermal variation of carrier mobility within a temperature domain of 100–500 K (Figure 6d,e) indicated that this mobility’s thermal sensitivity is not contingent upon the presence of strain below 200 K. Between 200 to 500 K, mobility values for electrons and holes in unstrained BAs correspond to T^−2.14^ and T^−2.41^ in power law behavior, respectively. Conversely, under tension, these mobilities adhere to an approximate T^−1.8^ for electrons and T^−3.0^ for holes. Moreover, BAs showcases a near lattice match with InGaN and ZnSnN_2_ (Figure 6f) and is anticipated to engender a type II band alignment when interfaced with these materials. Therefore, employing BAs as a foundational platform portends its utility as an epitaxial base for layering materials like InGaN and ZnSnN_2_ to constitute p-n junctions apt for photovoltaic applications [59].

Rout et al. [60] performed an in-depth exploration of the impact that epitaxial strain exerts upon the electrical and optical behaviors of the nonconventional double perovskite, Ca_2_FeOsO_6_. Their research demonstrated a tunable critical temperature induced by the applied strain effects, whilst the spin coherence length significantly surpassed that observed in the well-known spintronic material, Sr_2_FeMoO_6_. The imposition of a +5% strain notably triggered a bandgap transition from indirect to direct, enhancing electron–hole pair disentanglement within confined states upon photon excitation. With its potential limiting efficiency reaching up to 33%, Ca_2_FeOsO_6_ stands out as a robust contender for harnessing solar power. Thereby, this compound presents substantial promise for future applications in the domains of spintronics and optoelectronics, where epitaxial strain acts as a pivotal factor in property modulation.

### 4.3. Power Degradation

Working in a high strain state for a long time, solar cells may experience the degradation of power output. This effect can be attributed to a variety of mechanisms, such as microcrack formation, interlayer stripping, and increased carrier recombination efficiency, which will weaken the overall performance of the battery. Understanding the mechanism behind solar cell device instability is crucial for advancing photovoltaic technology [62].

**Figure 7:**
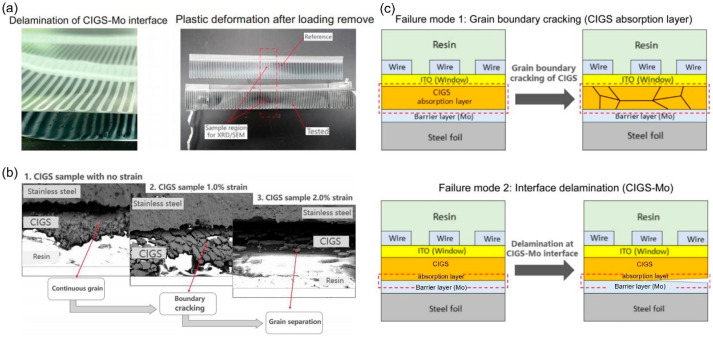
(**a**) A visual examination of CIGS solar cell specimens subsequent to a tensile test. (**b**) SEM analysis delineating the microstructure of CIGS at varied strain magnitudes. (**c**) Two discrete strain-induced failure modes witnessed in CIGS thin-film solar cells. (Reprinted with permission from Ref. [63]. Copyright 2020, Elsevier).

The effect of strain on the power consumption reduction in Cu (In,Ga)Se_2_ (CIGS) thin film solar cells has been studied in detail. The degradation of power is rooted in two primary mechanisms. Initial observations indicated that when tensile strain did not exceed the 1.5% threshold, it led to the fracturing of grain boundaries within the CIGS absorptive layer, compromising carrier transport and recombination along these boundaries. Further, when the tensile strain escalated to 2.0%, shear stress incited the detachment at the CIGS-Mo interface (Figure 7a), disrupting the integrated contact with the backing electrode. Figure 7b illustrates the morphologies imaged by SEM for samples subjected to varying strains. Figure 7c shows the mechanisms behind the power degradation in CIGS thin-film cells, attributing it to alterations in microstructural integrity, with the emphasis that once degradation manifests, its reversal becomes a challenge. As such, it is necessary to delve deeper into the relationship between strain and power decline within CIGS thin-film solar cells [63].

### 4.4. Trap State Density

In solar cells, strain may also cause the formation of defect states, increasing the trap state density. These trap states are equivalent to non-radiative recombination centers, which weaken the collection efficiency of light-generating carriers and thus reduce the photoelectric conversion efficiency. Cheraghizade et al. [64] delved into the performance dynamics of TiO_2_/SnS/Ag and TiO_2_/CdS/SnS/Ag structured solar cells. The presence of a CdS layer enhances strain induction and trap state density in the solar cells, a result of lattice incompatibilities and differential thermal expansion behaviors. It was observed that elevated operational temperatures attenuate trap state densities, which in turn augments the mobility within the solar cell and lowers the threshold voltage. The observed phenomena might stem from enhanced passivation of interfaces and defects along with the restriction or localization of trap states through thermally driven ionization at the core of traps. Such experimental insights are crucial for grasping the photovoltaic response of alternative third-generation solar cell materials.

### 4.5. Ion Migration

Ion migration within perovskite thin films tends to engender adverse band bending, interface reactions, and phase segregation, consequently impairing PSC operation [65].

This ion movement constitutes a significant factor underlying the decline in perovskite solar cells’ efficiency. Zhao et al. [66] analyzing the impact of divergent interstitial cations on iodide ion migration energies toward proximal vacancies within perovskite structure, unveiled the greatest VI migration energy barrier (2.80 eV) in systems incorporating Nd^3+^. They postulated that such disparities in ion migration resistance could be ascribed predominantly to divergent cationic valence states. Concurrently, variations in doping concentrations associated with optimal power conversion efficiencies were found to be cation-dependent. The use of Nd^3+^ dopants corresponded well with J-V scan data, yielding notably stable PCE values. A doping rate of 0.08% Nd^3+^ resulted in film surfaces congruent with reference samples and maximized energy interactions between the defect and Nd^3+^, thus potentially reducing side effects. Furthermore, Nd^3+^ doping boosts photoluminescence lifespan, which supports the effectiveness of Nd^3+^ dopants in reducing defects. Exploring defect density variations in films doped with different cations unveiled reduced negative charge or neutral defects within the bulk region due to doping. Additionally, in situ PL observations under a 440 nm beam and an applied field of 150 mV/μm revealed substantial inhibition of charged defect migration upon introducing 0.08% Nd^3+^. Such findings bolster PSC advancement.

### 4.6. PCE and Stability

During high temperature annealing, the residual tensile strain in α-FAPbI_3_ perovskite films can cause phase transition, which seriously affects the stability of α-FAPbI_3_. The residual strain of FAPbI_3_ thin films was released by using MASCN. After MASCN surface treatment, the average PSCs efficiency of FAPbI_3_ increased from 18.96% to 20.83%. In the humidity stability test, the FAPbI_3_-MASCN device still maintains the initial efficiency of 88.6% after 1000 h, while the ordinary device can only maintain the initial efficiency of 54.1%. In the thermal stability test, the FAPbI_3_-MASCN PSCs maintained an initial efficiency of 87.1% after annealing for 1000 h, while the ordinary device only maintained an initial efficiency of 66.9%. After 1000 h of optical aging, the initial efficiency of the ordinary device was only 25.5%, while the FAPbI_3_-MASCN device maintained an initial efficiency of 88.5%. In addition, the 15.32% PCE achieved on PSCs modules with an effective area of 9.6 cm^2^ is significant for large-scale manufacturing of PSCs [67]. The instability of perovskite/silicon tandem solar cells remains one of the key obstacles to practical application, which is closely related to the strain of perovskite thin films. A strain-free perovskite film was obtained by post-treatment with a mixture of N,N-dimethylformamide and n-butylammonium iodide in isopropanol solvent. This reduces the defect density and inhibits ion migration. The corresponding single-junction perovskite solar cells had a PCE of 21.8% (higher than the untreated 18.7%), while the unencapsulated devices PCE retain 100% and 81%, respectively, after storage in N_2_ and air for more than 2500 and 1800 h, respectively [68]. The quality of perovskite films is crucial to the realization of high-performance PSCs. The soft perovskite–substrate interface constructed with amphiphilic soft molecules (ASMs) with long alkyl chains and Lewis bases is very effective in reducing interfacial strain. The PCE of the optimized device was 19.7%, while that of the untreated one was only 17.3%. Even more impressively, it was continuously illuminated for more than 6200 h at high temperatures (≈65 °C), maintaining 84% of its initial efficiency, compared with only 43% of that in reference [69].

By using the drift diffusion model, Meng et al. [53] proved that the strain-induced bandgap gradient increases with the increase in defect density. Under 1 solar condition, the defect state was achieved and the device performance of the strained and unstrained perovskite solar cells was found to be comparable. However, the output power density of the devices with strain under low light conditions is 20% lower than that without strain. They successfully demonstrated that stress has a significant effect on environmental stability. Cracks in perovskite films during thermal cycling greatly affect their stability. Yuan et al. [70] used compressive strain to suppress cracks and the delamination of polymers with low elastic modulus. The resulting device retained 95% of the initial PCE and compression strain after 230 cycles. At the same time, the highest PCE of the p-i-n device was 23.91%. This greatly facilitated the development of mechanically stable perovskite solar cells.

In general, the effect of uniform strain on the dark I–V characteristics of solar cells is reversible and more pronounced in thinner crystalline silicon solar cells, which should be further explored. In high-voltage batteries, matching NPL-sensitized cells with suitable high-voltage electrolytes can result in near-perfect short-circuit currents and internal quantum efficiency. The relationship between strain and power decline in CIGS thin film solar cells should be further studied.

## 5. Adjusting Method for Stabilizing Solar Cell Strain

This part mainly reviews local strain regulation (local relaxation, lattice strain reduction, residual stress release, etc.), externally induced strain regulation (material, temperature, pressure, etc.), and strain modulation strategies.

### 5.1. Local Strain Regulation

Local strain considerably influences the vacancies, electronic states, and stability of ABX_3_ perovskite structured materials. Ion replacement offers an enticing avenue for mediating crystallization states or facilitating electron presence in organic–inorganic hybrid halide lead perovskites. A moderately zinc-substituted regulatory ABX_3_ compound has recently been synthesized, achieving an orderly, robust CH_3_NH_3_(Zn:Pb)I_3_−_−x_Cl_x_ crystal by mitigating lattice strain through proper contraction within the BX_6_ octahedral configuration. Utilizing a CH_3_NH_3_(1Zn:100Pb)I_3_−_x_Cl_x_ binary metal perovskite has garnered a PCE value of up to 20.06% under akin-to-sunlight conditions, showcasing considerable stability. By comprehending the substitution effects and particulars of crystal growth, enhancements in both film quality and device efficacy can be pursued, with further crystal structure and compositional optimizations currently underway [71].

**Figure 8:**
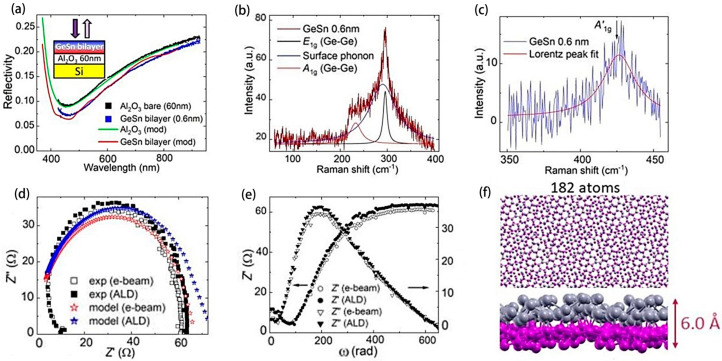
(**a**) The optical reflectance of an Al_2_O_3_ substrate with a thickness of 60 nm and a subsequent GeSn bilayer deposition is measures. The emissivity values for the bare substrate and the GeSn bilayer are represented in green and red, respectively. (**b**) An estimation of the Ge and Sn layer thicknesses, being approximately 0.3 nm, where the longitudinal mode E_1g_ and the transverse mode A_1g_ are discernible. (**c**) A′_1g_ in the GeSn bilayer fits well to the Lorentz form (red). (**d**) Nyquist plot: experimental values of Z″ against Z′ (square). (**e**) The real and imaginary parts of the complex impedance Z′ (circle) and z″ (triangle) are taken as a function of the frequency ω = 2πf. (**f**) A top view of a single cell, disclosing lattice distortions in not just the XY-plane but also in the Z direction. (Reprinted with permission from Ref. [73]. Copyright 2023, Springer Nature).

The dual substitution of the FA site in FAPbI_3_ with equimolar proportions of cesium (Cs) and methylenediamine (MDA) cations was surprisingly effective. It was found that the presence of MDA and Cs cations at 0.03 molar fractions alleviated lattice strain, boosted carrier longevity, diminished Urbach energy, and curtailed defect concentrations. Subsequently, the reduction in non-radiative carrier recombination facilitated certified efficiency rates at 24.4% for small PSCs and 21.6% for larger 1 cm × 1 cm cells, with these devices also demonstrating exceptional thermal stability [72]. Zhang et al. [73] presented a dual-layer construct replacing traditional van der Waals forces between layers with stronger covalent bonds. As an exemplar, GeSn bilayers were substantiated via ellipsoid reflectance (Figure 8a), Raman spectroscopy (Figure 8b,c), and impedance spectroscopy (Figure 8d,e). The impedance analysis elucidated significant capacitive behavior with a capacitance of 102 μF and a dipole charge density ranging from 1–2 × 10^−4^ μC/cm^2^ at the bi-oxide interface. It is inferred that these attributes may relate to lateral strains induced by the substrate and the buckling inherent to the bilayer’s form. In low-twist-angle GeSn bilayers, strain distortions give rise to a plethora of physical occurrences associated with topological defects (Figure 8f). Such discoveries present extensive potential for subsequent innovations in solar technology and energy domains.

Juxtaposing this advanced material research, the performance of PSCs, as it approaches commercial viability, is hindered by stability concerns and the challenges posed by residual strain and their adverse effects on PSC photovoltaic efficiency. Through the creation of perovskite films with varied spacer cations, the researchers effectively harnessed the tensile strain, optimizing the film crystalline structure. The released strain facilitated charge movement, minimized recombination, and the practical result was an improved PCE. Additionally, these strain-alleviated devices displayed superlative stability, preserving their initial efficiency following prolonged periods of storage and thermal testing, marking a step forward in the deployment of perovskite-based optoelectronic devices [74]. Moreover, the persistent challenge of lattice strain in halide perovskites poses significant threats to the efficiency of PSCs. This can be addressed by supplementing the organic ammonium halide after thermal annealing to mitigate the residual strain in the film. Post-processing treatment allowed for the alleviation of residual strain, promoting crystallinity and repairing imperfections. The combined effect of this compositional compensation and strain relief yielded PSCs devoid of strain, achieving a PCE of 21.30%, with remarkable stability of 98% efficiency retention at 45 °C after 1000 h period. This development is a boon for the industrial application of PSCs [75].

Optimizing the microgeometry of perovskite films maximizes photovoltaic performance and extends durability. By tailoring the solubility temperature relationship between dimethyl sulfoxide and ether the control the morphology of micro-wrinkles in thin films. Lowering the ether temperature led to the slender viscoelastic layer, covered by a relatively stiff film, relaxing the compressive stresses, resulting in pronounced wrinkling. They observed that the wrinkled configuration developed at 5 °C exhibited a superior PCE and enhanced stability compared to the planar formation at 30 °C. The findings indicated that sites with larger fold amplitudes reduced defects propelled the carrier lifetime extension and bolstered the overall photo-response and *V*_OC_. This investigation elucidates the underlying association between lattice strain and carrier dynamics in PSCs [76].

### 5.2. External Conditions Induce Strain Regulation

The presence of both tetragonal and cubic phases in perovskite layers exerts strain on the crystal structure and contributes to defect formation, adversely affecting photovoltaic performance and device stability. Ye et al. [77] illustrated the formulation process for a strain-free MAPbI_3_ film in mesoporous solar cells. By inserting a lattice-matched two-nanometer thick CsPbBr_3_ buffer layer acting as a growth template for MAPbI_3_, the relaxed growth of RP layer was achieved. Post-optimization, PCE values for solar cells rose to 22.12%, with marked enhancements in environmental, light, and thermal stability, reinforcing that crystal strain tuning could forge high-performance PSCs.

**Figure 9:**
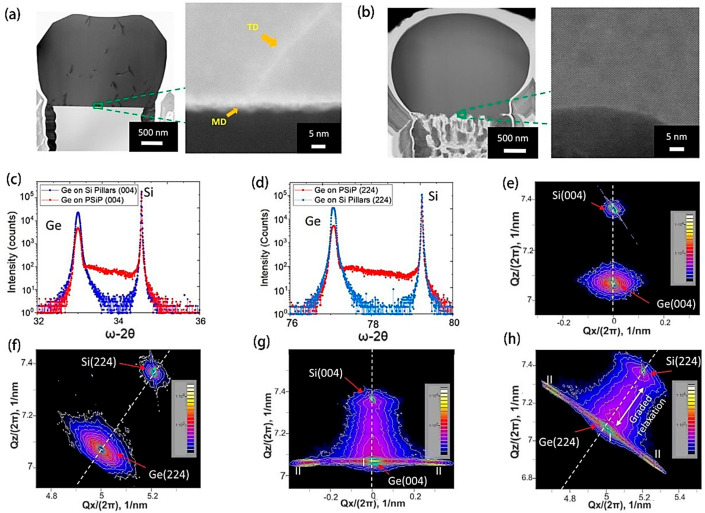
(**a**) Bright field transmission electron microscopy (BF-TEM) images were used to characterize Ge/SiP reference substrates, as well as high-resolution STEM images showing thread dislocations and mismatched networks at Ge/Si interfaces. (**b**) BF-TEM images showed defectless growth of germanium on porous silicon columns (PSiP), and atomic resolution STEM imaging showed Ge/PSiP interface structure. (**c**) Ge/SiP and (**d**) Ge/PSiP(70%) ω-θ around Si(004) and (224). Ge/SiP reciprocal spatial mapping of symmetric (**e**) Si (004) and asymmetric (**f**) Si (224). Reciprocal spatial mapping of Ge/PSiP heterostructures around symmetric (**g**) Si (004) and asymmetric (**h**) Si (224) reflections. According to their structural characteristics, germanium microcrystals can be divided into two categories: relaxation (I) and asymmetric relaxation (II). (Reprinted with permission from Ref. [78]. Copyright 2022, Springer Nature).

Recently, the synthesis of fully flexible substrates for the epitaxial growth of lattice mismatch materials on silicon was developed. The employment of in-depth patterning and electrochemical porosity contributed to the formation of silicon pillars, resulting in a reduced Young’s modulus and thus making it easy to deform to accommodate lattice mismatches. Ge microcrystals cultivated on SiP exhibited an elevated defect density (Figure 9a). Conversely, Ge microcrystals that were developed on porous silicon phosphide (PSiP) demonstrated an absence of defects, which is clearly shown in Figure 9b. The enhancement in the quality of Ge crystals when grown on porous substrates, along with the achievement of gradient relaxation among the materials, is corroborated by high-resolution X-ray diffraction and reciprocal space mapping focused on both symmetric silicon (Si) (004) reflections and asymmetric Si (224) reflections (Figure 9c–h). These findings underscore the effectiveness of employing porous media in the cultivation of Ge microcrystals for the significant reduction in defect densities, thereby facilitating a better interface between the composite materials [78].

Within the realm of all-inorganic perovskite solar cells featuring an active layer composed of CsPbI_3_, Yu and colleagues [79] demonstrated the use of organosilazane (OPSZ) as a tensile strain-regulating interlayer introduced during the annealing process. The excellent solubility of OPSZ in antisolvent environments facilitates efficient alteration of the perovskite layer’s surface properties. Notably, altering the annealing temperature of the OPZS solution from 25 °C to 75 °C, and subsequently to 125 °C, exhibits a marked progression from tensile to neutral and then to compressive strain within the material. The augmented mechanical resilience of the post-annealed OPSZ strain-compensating layer bestows upon the cells enhanced durability against storage and light-induced degradation. Indeed, devices incorporating this layer maintained 90% of their initial efficiency after a decade-long storage period and preserved 95% efficiency following four days of continuous exposure to light. Furthermore, OPSZ treatment has been shown to enhance the crystallinity of the films, reduce trap densities, and inhibit non-radiative recombination pathways. As a result, the PCE of the finely tuned PSCs reached a zenith of 19.12%. The study underscores the crucial role of strain engineering in boosting the performance of inorganic PSCs. Moreover, the application of compressive strain via the hole transport layer (HTL) offers a method to counteract thermally induced tensile strain in the perovskite film. Through the precise regulation of the processing temperatures for HTLs with substantial thermal expansion coefficients, researchers have been able to fabricate perovskite films with adjustable strain characteristics. The introduction of compressive strain raises the activation energy necessary for ionic migration, thereby enhancing the overall stability of the films. Experimental results demonstrate that PSCs benefiting from compressive strain exhibit high operational efficiencies even at raised temperatures, marking them as amongst the most stable wide-bandgap perovskite materials identified to date [80].

The incorporation of paracetobromide (AABr) into the crystal lattice of MAPbI_3_ has led to the development of highly efficient and durable PSCs. Computational studies using density functional theory (DFT) reveal that AABr enhances the structural integrity of MAPbI_3_ thin films through the formation of NH-I hydrogen bonds, which alleviate tensile strain. Furthermore, AABr serves as a suppressor of charge-carrier recombination, reducing defects at the interface of materials and thus enhancing charge collection efficiency. Researchers have observed that solar cells treated with AABr within MAPbI_3_ achieve a PCE of 20.18% and demonstrate significant longevity even without encapsulation [81]. In related research, Nishimura et al. [82] investigated the correlation between lattice strain and solar cell performance. They determined that reducing lattice strain within the compound Q_0.1_(FA_0.75_MA_0.25_)_0.9_SnI_3_—where Q denotes cations like Cs^+^, K^+^, Na^+^, butylammonium^+^ (BA^+^), and ethylammonium^+^ (EA^+^)—leads to an enhancement in solar cell efficiency, especially as the tolerance factor approaches one. The specific formulation EA_0.1_(FA_0.75_MA_0.25_)_0.9_SnI_3_, which showed minimal lattice strain, achieved the highest efficiency at 5.41%. Moreover, a consistent improvement in the performance of SnGe-PSCs, from 6.42% to 7.60%, was noted over storage periods. This increase is attributed to the relaxation of lattice strain, suggesting potential strategies to optimize the performance of Sn-based PSCs.

### 5.3. Strain Modulation

**Figure 10:**
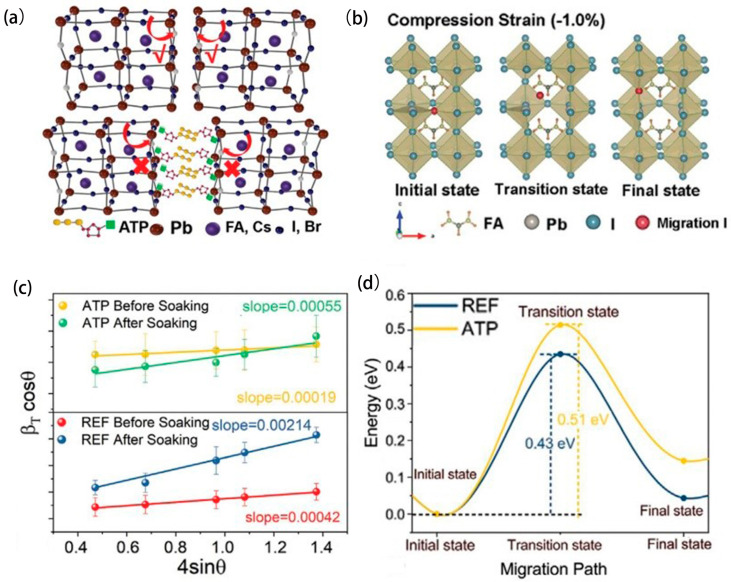
(**a**) Schematic diagram of the interaction between perovskite and ATP. (**b**) The ion migration scheme in the compressed strain lattice. (**c**) W-H diagrams of REF and ATP. (**d**) Strain activation energy of ion vacancy assisted migration in ATP and REF films. (Reprinted with permission from Ref. [83]. Copyright 2022, Wiley Online Library).

As of now, considerable research has been conducted on the application of strain engineering in individual-junction PSCs. However, there have been few studies concerning perovskite/silicon tandem devices. Recently, a newly developed strain regulation technology using adenosine triphosphate (ATP) has advanced the manufacture of durable perovskite/silicon tandem solar cells. X-ray photoelectron spectroscopy (XPS) investigations reveal that ATP can engage with Pb^2+^ more readily in comparison to the reference films. Figure 10a delineates a model elucidating how ATP prefers to localize at the grain boundaries within the perovskite matrix. Through the application of the W-H method, detailed in Figure 10c, the authors quantified the internal micro-strain of the films and ascertained that the ATP introduced a minimized micro-strain at all stages, including both fabrication and aging phases. The authors hypothesized that ATP’s presence effectively transforms residual tensile strain into a compressive state within the perovskite absorbers. The mechanism for ion migration when subjected to compressive strain is demonstrated in Figure 10b, and its implications are substantiated in Figure 10d, where reduced ion migration is evidenced in ATP-incorporated films. The researchers successfully produced an efficient n-i-p structured single perovskite/silicon series solar cell, achieving an impressive PCE of 26.95% [83]. The distribution of strain in the device can also be regulated by changing the substrate. Park et al. [84] prepared a new transparent electrode of F-PSCs using a colorless polyimide (CPI) substrate. The mechanical flexibility of the optimized device is significantly better than that of conventional samples because the CPI substrate reduces the strain generated during internal and external bending. After 100 bending tests, the CPI-based device retained more than 80% of the initial PCE. The development of transparent electrodes facilitated the realization of translucent F-PSCs, which is expected to realize its value in smart displays and windows. The flexibility of F-PSCs makes it widely used in wearable electronic devices, new energy vehicles, and smart buildings [85].

Overall, the newly developed fully flexible substrate for the epitaxial growth of lattice mismatched materials facilitates the matching of composite materials, which will provide a new method for the production of large-area, defect-free heterostructures. However, the effectiveness of flexible substrates in high strain environments and the strain-induced power attenuation need further study. Ion substitution provides an attractive way to regulate the crystalline state of materials or to promote the presence of electrons. But this may lead to changes in the crystal structure, which can affect the physical and chemical properties of the material. It is necessary to further study the effect of ion substitution on the structure and properties of materials, establish an accurate theoretical model, and increase the applicability of ion substitution. The combination of substitution effect and crystal growth optimization can pursue the improvement of film quality and device efficiency. Prospective investigations could integrate doping techniques with external pressure application to better improve the photonic and electronic band structures. Post-processing plays an important role in addressing the ongoing challenges of lattice strain on the efficiency and stability of PSCs by alleviating residual strain, promoting crystallinity, and repairing defects. Therefore, the method of combining defects compensation and strain mitigation should be further explored.

## 6. Strategies to Improve Solar Cell Performance

As previously indicated, the presence and magnitude of strain play critical roles in dictating the electronic band structure, optoelectronic characteristics, ion migration, and overall power output in solar cells. Consequently, strain engineering can exert either favorable or deleterious effects on the efficacy and durability of solar energy systems. The forthcoming discussion will center on methodological approaches leveraging strain engineering to augment solar cell performance [36].

In their seminal research, Chen et al. [86] delved into the strain’s influence on amorphous silicon and halide-PSCs. Diverse substrates, specifically fiber-reinforced polymers and glass, were employed to support these two solar cells. It was discerned that amorphous silicon solar cells showed signs of degradation when subject to compressive strains ranging from 0.5% to 1%, while PSCs manifested similar deterioration under tensile strains exceeding 3%. Solar cells are therefore existent to certain strain thresholds, within which they maintain optimal function. Surpassing these thresholds precipitates rapid performance decline. The difference is that the reason for the decreased performance of perovskite solar cells is the fracture of the glass substrate, rather than the solar cell itself. This finding suggests that PSCs could potentially excel in environments characterized by high strain, meriting the further examination of their behavior on flexible substrates.

### 6.1. Quantum Dots/Quantum Wells

The application of III–V compound semiconductors for QW and quantum dot (QD) technologies has yielded substantial contributions to the evolution of solar cell devices.

Intermediate band solar cells (IBSCs) can be constructed using superlattices composed of quantum dots. They utilized an InAs/GaAsN composition for the QD/barrier material to bolster the count of QD layers, thereby enhancing the potential for sub-bandgap absorption. Nitrogen dilution within barriers was found essential in maintaining quantum dot layer strain equilibrium. Nonetheless, introducing nitrogen to the InAs/GaAs QD matrix diminished its potential as an IBSC due to suboptimal bandgap configurations. Alternative approaches such as augmenting the spacer layer thickness or substituting nitrogen with phosphorus in InAs/GaAsN may offer viable solutions. The findings suggest that GaAsN dilution alloys may not be ideal candidate materials for IBSCs, and that while the initial intent for multi-layer InAs/GaAs QD-IBSCs with 1% nitrogen in the barrier, including strain balance formation to enhance QD layer stacking, was promising, it faced limitations due to an inability to recover the GaAs emitter voltage [87].

**Figure 11:**
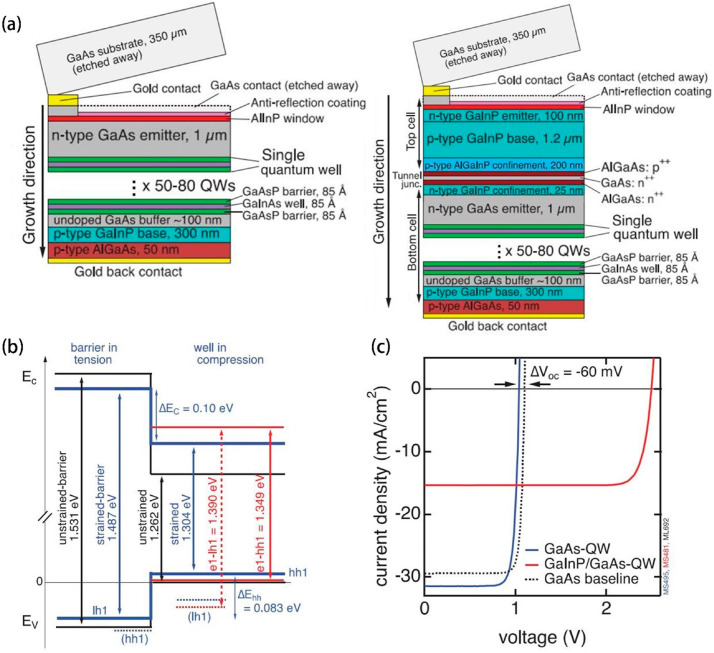
(**a**) Schematic diagram of the layer structure of a single-junction GaAs-QWs battery (left) and a double-junction GaInP/GaAs-QWs battery (symbols ++ indicates high levels of doping). (**b**) Energy schematic of QWs with strain equilibrium: the unstrained alloy is denoted by a black line, the effects of strain by a blue continuum, and the quantum confinement’s additional impact within the wells by a red continuum. Heavy holes (hh1) and light holes (lh1) are represented by a solid line, and the dashed line represents the carrier of the dark transition. (**c**) Current-voltage curve at 1000 w/m^2^. (Reprinted with permission from Ref. [90]. Copyright 2020, Wiley Online Library).

Nanostructured QWs have been identified as a significant contributor to the enhancement of photovoltaic device performance, specifically in the realm of III–V solar cells. Within the spectrum of AM1.5 illumination, it has been observed that nanostructured single-junction III–V solar cells have attained an efficiency surpassing 26%. This surge in performance is primarily due to the integration of a heterojunction that demonstrates low dark current and a superlattice structure incorporating strained quantum wells. Such an architecture promotes efficacious carrier separation and reduces radiative recombination, imperative for the advanced operation witnessed in multi-quantum well solar cells, particularly those with barriers thinner than 4 nanometers. The potential exists to further augment device efficiency through the incorporation of strategies for light harvesting and/or implementing partial strain compensation methods, with strained-quantum well superlattice solar cells poised as a viable embodiment of high-efficiency photovoltaics [88]. QWs play an important role in improving the performance of triple junction solar cells and partially resolving Boltzmann losses. The radiation limitation of QW solar cells at high concentrations allows the photon effect to be exploited to improve the efficiency of solar cells. The strain-induced anisotropic emission in the single-junction structure brings efficiency gains to the solar cell. In multi-junction structures, the use of radiation-efficient QWs allows excess energy to flow downward, enabling solar cells to actively adapt to current conditions [89]. In the seminal work, Steiner et al. [90] adeptly embedded strain-balanced GaInAs/GaAsP QWs within solar cells that employ either GaAs alone or a combination of GaInP/GaAs. These configurations demonstrated enhanced carrier collection capabilities and a significant reduction in non-radiative recombination instances. The figures below collectively show the GaAs-QW solar cell with an ingenious energy structural (Figure 11b) optimized for quantum well design and the associated single and dual-junction GaInP/GaAs-QW cells (Figure 11a), all exemplifying technological progress. Precision in strain balancing allows for the inclusion of numerous QWs without causing plastic relaxation, thus harnessing the superior photon absorption properties of high-quality GaInAs material, especially for energy below the GaAs band edge. The single-junction devices equipped with a refined heterojunction (RHJ) structure maintain an open circuit voltage of 1.04 V, notwithstanding the substantial quantum waves (Figure 11c). These innovations culminated in a record-breaking 27.2% efficiency for single-junction GaAs-QW solar cells and an unprecedented 32.9% for dual-junction GaInP/GaAs-QW cells. The benefits conferred by the addition of QWs to the bottom GaAs sub-cell were empirically confirmed. However, improving the FF in QWs-based solar cells remains an obstacle.

### 6.2. Improve the Efficiency and Stability of Photoelectric Conversion

**Figure 12:**
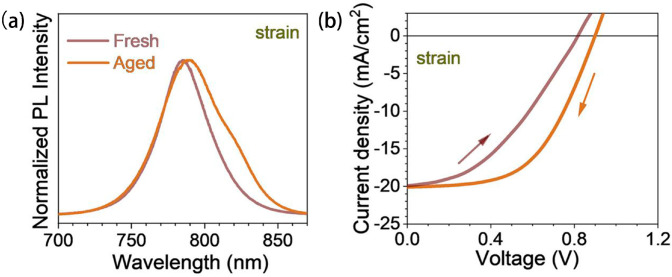
(**a**) Normalized PL spectra of strained perovskite films before and after aging. (**b**) J-V curves from forward and reverse measurements of the degraded device (The ascending arrow indicates forward scanning, and the descending arrow indicates reverse scanning). (Reprinted with permission from Ref. [53]. Copyright 2022, Cell Press).

In contemporaneous discourse surrounding solar cell device performance, the PCE is frequently cited as a pivotal metric, signifying the proportion of incident power (*P*_in_) converted into output power. Despite the monumental strides in augmenting both efficiency and durability in solar cells, the persistence of stability issues continues to throttle their mass-market adoption [91]. The operation of halide perovskite solar cells is strongly affected by ion migration. This is especially true when considering the long-term stability of the device [92]. This can be attributed to strain-induced defects at the interface. These defects are thought to trigger ion migration and phase separation and accelerate degradation. The photoluminescence (PL) spectrum of the continuously strained perovskite layer was broadened after continuous irradiation for 600 h. Hysteresis occurs (Figure 12a). When scanning in the opposite direction, the lag behavior for FF and V_OC_ is more obvious (Figure 12b). This study strongly demonstrates that interfacial strain defects play a key role in degradation by accelerating ion migration and phase separation [53]. Most literature studies have shown that tensile strain accelerates the degradation of PSCs. Moderate compression strain can improve the photoelectric performance and stability of PSCs. Therefore, the goal of strain regulation in PSCs should be to release the residual tensile strain and introduce controllable compressive strain as much as possible [36].

**Figure 13:**
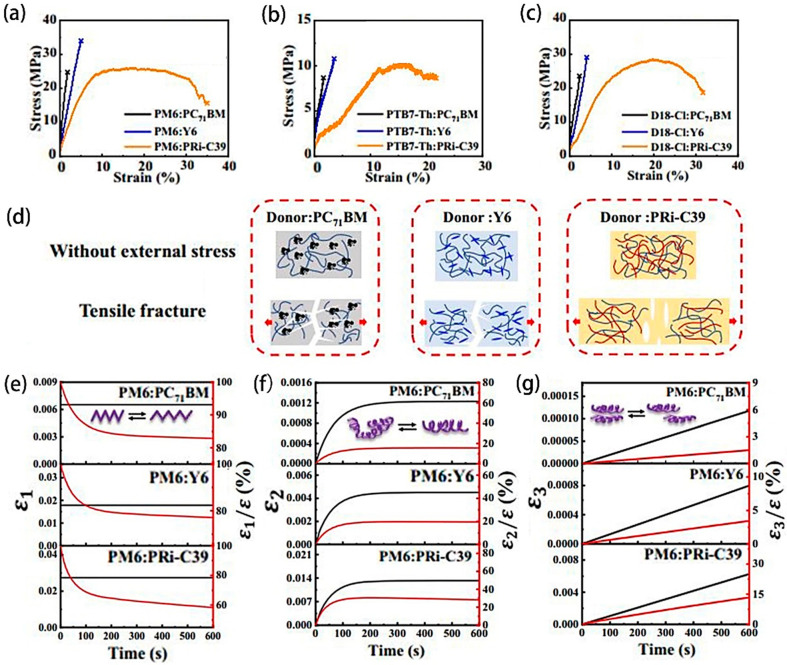
Displays the stress–strain profiles of (**a**) PM6-, (**b**) PTB7-Th-, and (**c**) D18-CI-derived mixed layers. (**d**) Film diagram without stress and fracture. (**e**) ε1, (**f**) ε2, and (**g**) ε3, along with their respective contributions to the total creep deformation over time. Illustration in (**e**–**g**): Schematic diagrams of molecular motion. (Reprinted with permission from Ref. [93]. Copyright 2023, Springer Nature).

The bulk heterojunction (BHJ) films’ mechanical attributes are seminal to the enduring stability of flexible-PSCs (F-PSCs). Zhong et al. [93] conducted an investigation into the multi-scale mechanical characteristics of BHJ films, incorporating three distinct electron acceptors. In the initial stage of linear growth, a successive decrease was observed in the slope of the curves for PC71BM, Y6, and PRi-C39 films measured using the FOW method (Figure 13a–c). Consequently, there was a corresponding decrease in the tensile modulus, while the fracture strain and toughness exhibited incremental value. The stronger interaction between the donor and Y6, as well as the entanglement with the donor, were visually evident in the pre-stretching and fracture behavior displayed in Figure 13d. Notably, Y6 demonstrated a more robust interaction with the donor and reduced entanglement compared to other electron acceptors. Furthermore, the time-dependent creep analysis derived from macroscopic tensile tests and microscopic indentation features (Figure 13e–g), indicated that PC71BM effectively reinforced the BHJ interpenetrating networks and minimized creep development. Additionally, the results of the contact angle test underscored the varying degrees of interaction between PM6 and the receptor, elucidating its influence on the mechanical properties of the membrane. This comprehensive study contributes significantly to enhancing the stability of F-PSCs.

In a groundbreaking study, a groundbreaking interlayer material known as Zn^2+^ chelated polythylenimide (PEI-Zn) demonstrated remarkable advancements in ultra-flexible organic solar cells (OSC). The research achieved a noteworthy PCE of 12.3% and 15.0% when utilizing two different electrodes (PEDOT: PSS and AgNWs) in ultra-flexible non-fullerene solar cells featuring PEI-Zn intermediate layers. The analysis based on the resistance relationship and the bending strain equation (ε = Hs/(2r), where Hs represents the thickness of the PES substrate) indicated that the PEI-Zn intermediate layer exhibited a bending strain capacity that exceeded twice that of ZnO. Remarkably, SEM images of PEI-Zn films revealed no cracks even after being subjected to 100 continuous bending cycles. Further mechanical robustness evaluations were conducted using pre-strained elastomer VHB, showcasing the superior mechanical properties and mechanical robustness of the PEI-Zn ETL-based battery compared to its ZnO counterpart. Upon subjecting the battery integrated with the PEI-Zn ETL to 100 consecutive compression–flat deformation cycles, its power conversion efficiency remained nearly constant [94].

**Figure 14:**
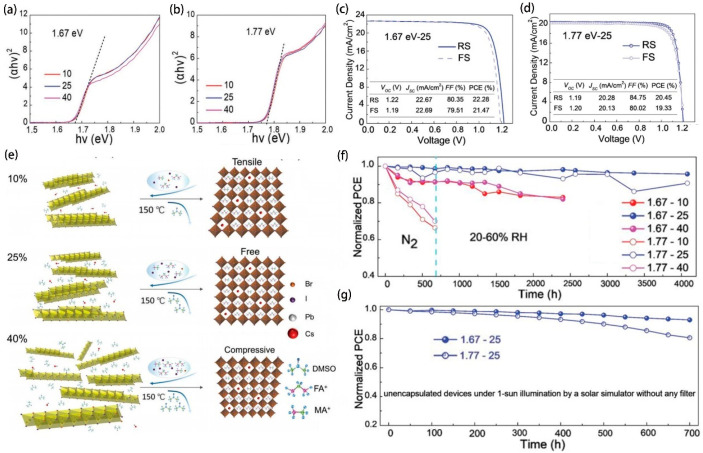
The UV-VIS spectra of perovskites with varying DMSO concentrations exhibiting bandgaps of (**a**) 1.67 eV and (**b**) 1.77 eV. The J-V characteristics for the devices, representing perovskites with bandgaps measuring (**c**) 1.67 eV and (**d**) 1.77 eV, each incorporates a 25% concentration of DMSO. (**e**) Schematic illustration elucidates the mechanism underlying strain formation in the perovskite structure. (**f**) Damping stability assessment of the apparatus in a lightless environment. (**g**) PCE tracking for a strain-free device MPP with two bandgaps in N_2_ atmosphere. (Reprinted with permission from Ref. [96]. Copyright 2023, Wiley Online Library).

The synthetic compound Spiro-OMeTAD HTL has been documented to accelerate the deterioration of CsPbI_2_Br perovskite structures, where residual thermal tensile stresses notably compromise the material’s stability. In an innovative approach, the study employed the semiconductor qualities of PCPDTBT, notably its P-type conduction, as a replacement for Spiro-OMeTAD to fulfill dual roles—mediating hole transport and modulating strain. Distinctively, the undoped PCPDTBT exhibits admirable capabilities in hole extraction and conduction, while concurrently elevating the humidity robustness of the perovskite. With cooling to ambient temperatures, PCPDTBT’s innate compressive strain has been shown to counterbalance the extant tensile discrepancies within the perovskite, thereby augmenting its stability to light exposure and thermal conditions. This investigation delineates an opportunely direct and effectual methodology for the cultivation of efficacious and persistent CsPbI_2_Br-based PSCs [95]. In parallel research, Hang et al. [96] delved into a simplistic yet effective technique for modulating strain during the fabrication of high-performing, stable wide bandgap (WBG) PSCs. Their exploration into the role of dimethyl sulfoxide (DMSO)—integrated at differential constitutions of 10%, 25%, and 40%—revealed its preponderant impact on the strain-influenced crystalline evolution process of WBG perovskite during sequential depositing stages. The strategic reduction in strain culminated in the production of two distinctive WBG perovskite films, each characterized by bandgaps measuring 1.67 eV and 1.77 eV (Figure 14a,b). The derived PSCs manifested remarkable efficiency rates of 22.28% and 20.45%, respectively (Figure 14c,d). Figure 14e illustrates the underlying mechanics of strain genesis in the perovskite context. These PSCs attained in excess of 4000 h of storage persistence and surpassed 700 h of maintained maximum power point (MPP) output, even under unpackaged and continual full-spectrum illumination (Figure 14f,g). It is, however, pertinent to note that persistent stress within the perovskite may trigger severe photoinduced phase segregation, potentially undermining device integrity during protracted light exposure. 

The large *V*_OC_ loss results in the PCE of tin-lead PSCs generally being lower than that of lead cells. An additive 2,6-diaminopyridine (TNPD) was proposed to correct this problem. The integration of TNPD, with its efficacious pyridine moieties, into the FA_0.7_MA_0.3_Pb_0.5_Sn_0.5_I_3_ precursor solution, effectively mitigates the oxidation of Sn^2+^, thereby curbing the occurrence of p-doping level defects and consequentially augmenting the *V*_OC_. TNPD acts as a nucleating agent, fostering the growth of tin and lead perovskite crystals, facilitating the formation of uniform large-grain films devoid of pinholes, while also alleviating micro-strain engendered during film growth. In contrast to the control group lacking TNPD, the PCE exhibits a nearly 20% enhancement. This investigation provides a comprehensive molecular design blueprint for future endeavors in realizing efficient and robust Pb-Sn PSCs [97].

In general, solar cells possess a definitive strain capacity threshold, which when adhered to, ensures peak performance. Surpassing this critical threshold precipitates a swift decline in overall efficiency. Consequently, mastering strain management with these devices is a pressing concern that demands immediate attention. The strategic implementation of nano-engineered quantum wells for strain modulation has yielded remarkable advancements in photovoltaic efficacy. Such strained-quantum well superlattice solar cells can generate higher short-circuit currents and open-circuit voltages, causing this type of solar cell to be at the forefront of application in restricted emission photovoltaic devices. However, ameliorating the FF of QW-based solar cells presents a formidable challenge, and a target FF value of approximately 85% would signal a significant milestone.

## 7. Outlook

This review provides a meticulous and comprehensive exposition of the various applications of strain engineering to photovoltaic technology, with a particular focus on solar cells. Initially, this paper categorized and delineated the types of strain—induced by lattice mismatches and external environmental influences. Concomitantly, it sought to unravel the mechanisms underpinning strain propagation and ascertain its effects on the optoelectronic properties of solar cells through sophisticated characterization methodologies. The discussion then pivoted to the influence of strain imposition on solar cell property and elucidated methods for strain stabilization. Finally, it systematically presented a strategy for improving solar cell efficiency through strain engineering.

Despite the significant advancements achieved in solar cell technology in recent times, a myriad of challenges stubbornly remain unaddressed. The forward march of progress demands the judicious application of strain engineering as a tool to facilitate the production of premium-quality solar cells. This necessitates intensified research into the origins of strain. Although existing scholarly endeavors have illuminated the strain emergent from the lattice dynamics of MHP configurations, provoked by heteroepitaxy, alongside the intrinsic crystalline architecture of perovskite films, the mitigation strategy of strain increase during crystal maturation needs to be explored. Moreover, the fine-tuning of substrate compositions to modify lattice constants presents a tantalizing avenue for future research. Techniques like transient reflection spectroscopy offer profound insights into the intricacies of internal strain dynamics within two-dimensional perovskite single crystals. The nanoindentation technique plays an important role in the exploration of flexible thin film photovoltaic technology. While the bandgap modulation induced by interfacial strain holds promise for enhancing device stability, it remains essential to consider the resulting increase in defect density. Thus, future investigations should integrate doping techniques with external pressure applications to optimize the photonic and electronic band structures. Modulating the critical temperature via strain adjustments also emerges as a viable strategy to bolster solar cell photoelectric parameters. The advent of quantum superlattice configurations has notably elevated solar cell efficiencies, thus presenting expansive vistas for research in quantum structure-based (QWs and QDs) solar cell manufacturing. Nevertheless, while PSCs have demonstrated their adaptability within high-strain environments, the implications of such conditions on their efficacy when applied to flexible substrates and the phenomenon of strain-induced power decay necessitate further investigation. In sum, strain engineering represents a pioneering framework that greatly improves the overall performance of solar cells, meriting profound expansion in future research endeavors.
